# Effects of Alkaline-Earth-Metal Ion Co-Doping in Cerium-Doped Tl_2_LiYCl_6_ Crystals

**DOI:** 10.3390/molecules31203623

**Published:** 2026-10-11

**Authors:** Rastgo Hawrami, Elsa Ariesanti, Jarek Glodo, Lakshmi S. Pandian

**Affiliations:** 1 Physics Division, Life and Physical Sciences Department, Fisk University, 1000 17th Avenue North, Nashville, TN 37208, USA; 2 Radiation Monitoring Devices, Inc., 44 Hunt St., Watertown, MA 02478, USA

**Keywords:** dual-mode scintillation detectors, gamma-ray detectors, neutron detectors, thallium based elpasolite crystals, co-doped scintillator crystals

## Abstract

Cerium-doped Tl_2_LiYCl_6_ (TLYC) crystals co-doped with alkaline earth metal ions (Mg^2+^, Ca^2+^, Sr^2+^, Ba^2+^) are grown using as-received chemicals as ∅16 mm boules using the Bridgman method. Samples processed from each boule are characterized by their scintillation detector properties, such as energy resolution, decay time, and proportionality. More than 12% improvements in light yield are calculated for TLYC co-doped with Mg and Ca. Gamma-neutron pulse shape discrimination using a moderated AmBe neutron source with TLYC crystals co-doped with Mg and Ca result in figure-of-merits of 1.5 and 1.6, respectively.

## 1. Introduction

Materials research continues through the investigation of new compositions and material engineering for elpasolite crystals, which are double-halide compounds with the general formula A_2_BB′X_6_ (a face-centered cubic structure related to perovskites), and a handful of them—most notably cerium-doped Cs_2_LiYCl_6_ (CLYC) [1,2,3,4,5], Cs_2_LiYBr_6_ (CLYB) [2,3,6,7], Cs_2_LiLaCl_6_ (CLLC) [4,8,9], and Cs_2_LiLaBr_6_ (CLLB) [4,10,11,12]—have become important scintillator materials because they combine gamma-ray spectroscopy with thermal neutron detection in a single crystal. To achieve higher detection efficiency, recently, Tl ion substitution in several Cs-based materials, including elpasolites, have been investigated. In particular, substitution of Cs with Tl in Cs_2_LiYCl_6_ (CLYC) has led to the creation of Tl_2_LiYCl_6_ (TLYC), which has increased the effective atomic number Z_eff_ from 45 to 69 and the density from 3.3 to 4.5 g/cm^3^ [13,14,15,16,17,18,19].

Co-doping techniques have been used to improve crystal quality and performance. A previously published combinatorial approach showed that doped bulk scintillator materials can be rapidly optimized for their properties through concurrent extrinsic doping/co-doping strategies [20,21]. The following paragraphs highlight a few cases in which co-doping has modified the scintillation and physical properties of well-known scintillators.

CsI, CsI:Tl, and CsI:Tl,Bi single crystals were grown using the vertical Bridgman method, and the effect of Bi^3+^ co-doping on the CsI:Tl X-ray-induced afterglow was studied. The results showed a remarkable reduction in afterglow of about an order of magnitude, and in trap concentration, based on Bi co-doping—improvements related to the possible role of Bi in compensating for intrinsic nonstoichiometric defects [22]. The afterglow problem has prevented CsI:Tl single-crystal scintillators from being used for applications in the field of computer tomography and high-speed imaging. Yb^2+^ co-doping in CsI:Tl can reduce afterglow by at least one order of magnitude [23]. The composition was uncovered by simultaneously screening for a beneficial co-dopant ion among the alkaline earth metal family and by optimizing its concentration and that of Tl^+^ and Eu^2+^ ions. The composition with the best performance was identified as 0.1% mol Tl^+^, 0.1% mol Eu^2+^, and 0.2% mol Ca^2+^. This formulation shows an enhancement of energy resolution and light output at 662 keV, from 6.3 to 4.9%, and from 44,000 to 52,000 ph/MeV, respectively [23].

Another study showed that scintillation crystals can include a rare earth silicate, an activator, and a Group 2 co-dopant. In an iteration, the Group 2 co-dopant concentration may not exceed 200 ppm atomic in the crystal or 0.25 at % in the melt before the crystal is formed. The ratio of the Group 2 concentration/activator atomic concentration can fall in a range from 0.4 to 2.5. In another example, it was shown that the scintillation crystal could have a decay time of no greater than 40 ns, and in another example, it was shown that it could have the same or a higher light output than another crystal with the same composition but without the Group 2 co-dopant. Another example showed that, via co-doping, a boule can be grown crack-free [24]. Another study showed that a small cation could be introduced to intentionally form a positively charged interstitial to reduce the formation of halogen vacancies, which act as deep electron traps that promote undesirable afterglow [25].

Co-doping in CeBr_3_ and LaBr_3_:Ce alleviates the difficulties seen with the crystal growth of large, crack-free single crystals of these fragile materials. Aliovalent doping was employed to strengthen CeBr_3_ to reduce crystal growth constraints and improve ingot yields. In this study, six divalent (Ca^2+^, Sr^2+^, Ba^2+^, Zn^2+^, Cd^2+^, and Pb^2+^) and two tetravalent cations (Zr^4+^ and Hf^4+^) were investigated as dopants to strengthen CeBr_3_ without negatively impacting scintillation performance. Fluorimetry, preliminary scintillation, and preliminary fracture toughness measurements are presented for these aliovalent-doped scintillators. While Ca^2+^, Zn^2+^, Cd^2+^, and Hf^4+^ all exhibited little or no change in the peak fluorescence emission for 300 nm excitation, Sr^2+^, Ba^2+^, Pb^2+^, and Zr^4+^ exhibited varying degrees of red shifting. Also, Pb^2+^ had a drastic detrimental effect on scintillation. Initial micro-indentation data indeed indicate a noticeable increase in the fracture toughness of the doped crystals as compared to that of undoped CeBr_3_ [26,27].

The improved performance of large-sized Sr^2+^ and Ba^2+^ co-doped LaBr_3_:Ce^3+^ scintillation crystals has also been reported. The scintillation light output of both Sr^2+^ and Br^2+^ co-doped crystals have been significantly improved. Compared to 70,000 ph/MeV (at 662 keV) for Ce^3+^-only-doped LaBr_3_, Sr^2+^ and Ba^2+^ co-doping increases the light output by ~25% to 88,000 ph/MeV and 89,000 ph/MeV, respectively. The energy resolutions of both Sr^2+^ and Ba^2+^ co-doped crystals have been improved over a wide energy range as well. The scintillation decay time has been slightly lengthened with co-doping, with no observable secondary slow component. Co-doped crystals have exhibited very similar emission and excitation characteristics to the Ce^3+^ only crystal. Both Sr^2+^ and Ba^2+^ showed improved light output proportionality in the low-energy range [28]. Also studied were the effects of Li^+^, Na^+^, Mg^2+^, Ca^2+^, Sr^2+^, and Ba^2+^ co-doping on the scintillation properties of LaBr_3_:5%Ce^3+^. The values of light yield and energy resolution were derived for these iterations. Sr^2+^ and Ca^2+^ co-doped crystals showed excellent energy resolution compared to standard LaBr_3_:Ce; the proportionality of the scintillation response to gamma and X-rays of Ca^2+^, Sr^2+^, and Ba^2+^ co-doped samples also considerably improved [29]. Commercially available LaBr_3_:5% Ce^3+^ scintillators with photomultiplier tube readout generally showed an approximate energy resolution of 2.7%. By co-doping LaBr_3_:Ce^3+^ with Sr^2+^ or Ca^2+^, the resolution improved to 2.0%. Such an improvement is attributed to a strong reduction in the scintillation light losses that occur due to the radiationless recombination of free electrons and holes during the earliest stages inside the high-free-charge-carrier density parts of the ionization track [30,31]. This outlines a mechanism for this Ce-doped LaBr_3_ enhancement based on electronic structure calculations. This study also shows that Br vacancies are the primary electron traps during the initial stage of the thermalization of hot carriers, prior to hole capture by Ce dopants. Isolated Br vacancies were associated with deep levels, and Sr doping increased the Br vacancy concentration by several orders of magnitude [32,33]. Another group has also proposed scintillator materials based on crystalline alkaline-earth-metal halide crystals comprising at least one alkaline earth metal selected among Mg, Ca, Sr, and Ba being doped with at least one dopant that activates scintillation other than Sm^2+^, and being co-doped with Sm^2+^, with at least one halogen selected among Br, Cl, and I [32]. The scintillation physical process mainly depends on the dopant; as an example, Ce^3+^ substituting for the cation (La^3+^ and Y^3+^) provides fast, efficient emission, resulting in a fast decay time and yielding the most light under gamma detection.

The current work will present cerium-doped Tl_2_LiYCl_6_ (TLYC) crystals co-doped with alkaline-earth-metal ions (Mg^2+^, Ca^2+^, Sr^2+^, Ba^2+^), tracking their growth, sample processing, the scintillation performance of Ce-only doped and co-doped crystals, and well improvements in crystal quality and performance.

## 2. Results

### 2.1. Emission Spectra

**Figure 1:**
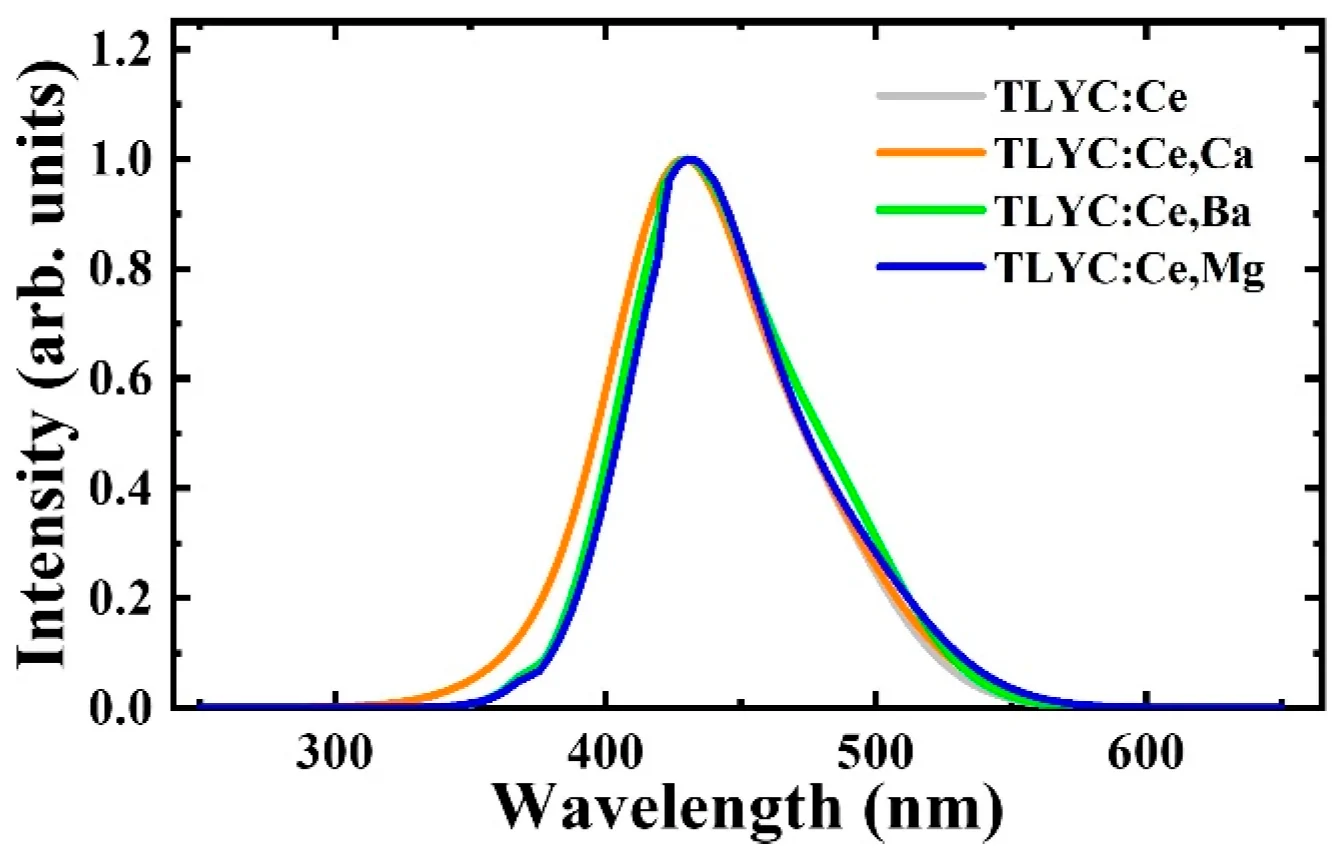
X-ray-excited optical luminescence spectra of TLYC:Ce, and TLYC:Ce co-doped with Mg, Ca, and Ba.

Figure 1 shows the emission spectra for TLYC:Ce co-doped with Mg, Ca, and Ba, compared to the emission spectrum of TLYC:Ce, as obtained using the X-ray-excited optical luminescence method or XEOL. The peak emissions for the observed spectra were similar in values: 429 nm for TLYC:Ce, 432 nm for TLYC:Ce,Mg, 428 nm TLYC:Ce,Ca, and 430 nm for TLYC:Ce,Ba. The co-doped TLYC crystals also had similarly broad emissions to those of TLYC:Ce, with the emission spectrum of TLYC:Ce,Ca being slightly expanded into the blue (UV) region and the emission spectra of TLYC:Ce,Mg and TLYC:Ce,Ba being slightly expanded into the red (IR) region.

### 2.2. Tl_2_LiYCl_6_:Ce,Sr

**Figure 2:**
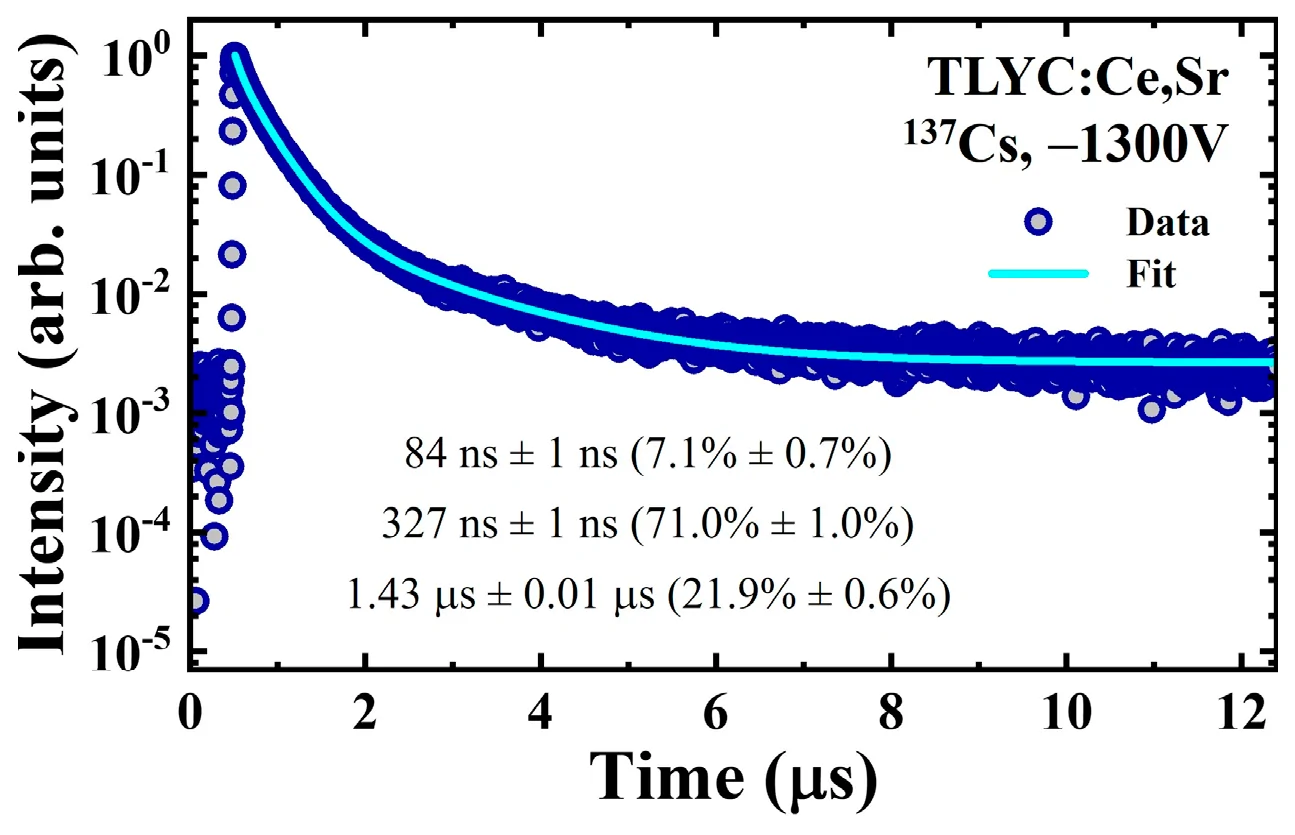
Temporal data of TLYC:Ce,Sr for decay time measurement.

The ^137^Cs spectrum was obtained with the TLYC:Ce,Sr sample; the spectrum was poor and appeared to show mostly photon scattering events, with no visible full-energy peak being resolved due to the quality of the crystal. Waveforms approximately corresponding to counts near the end of the spectrum were averaged, normalized, and then analyzed offline in OriginLab^®^ using the *ExpDec3* multi-component exponential function, which resulted in decay constants of 84 ns ± 2 ns (7% ± 2%), 327 ns ± 7 ns (71% ± 2%), and 1.43 μs ± 0.03 μs (22% ± 2%) (Figure 2).

### 2.3. Tl_2_LiYCl_6_:Ce,Mg

**Figure 3:**
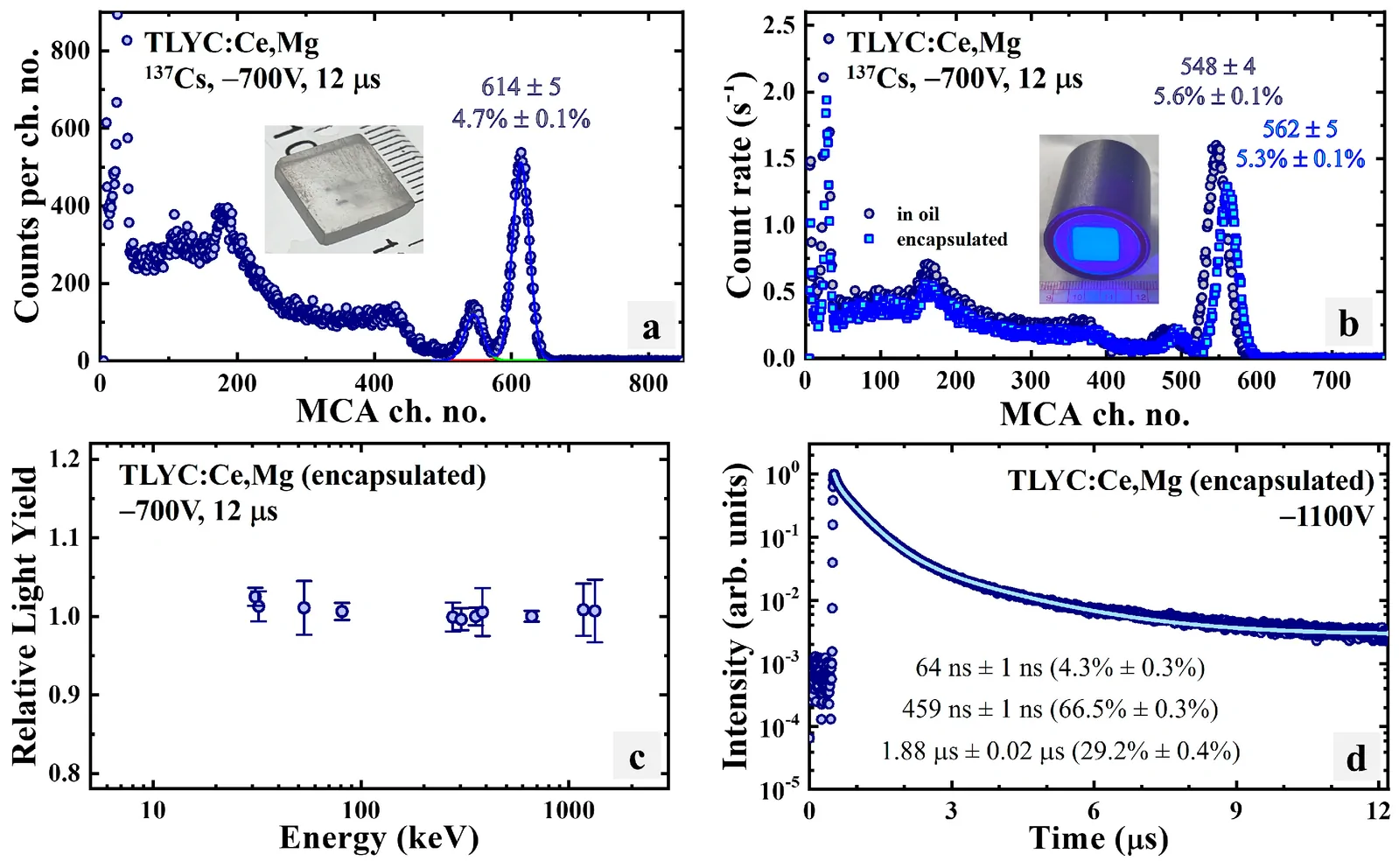
(**a**) ^137^Cs spectrum obtained with a 13 × 11 × 2 mm^3^ TLYC:Ce,Mg crystal. (**b**) ^137^Cs spectra collected by a TLYC:Ce,Mg crystal before and after encapsulation. (**c**) Non-proportionality data of TLYC:Ce,Mg. (**d**) Temporal data for TLYC:Ce,Mg.

Figure 3a shows the ^137^Cs spectrum obtained with a thin crystal of TLYC:Ce,Mg (approximately 13 × 11 × 2 mm^3^), with an energy resolution of 4.7% ± 0.1% (FWHM) at 662 keV. ^137^Cs spectra collected for a larger sized TLYC:Ce,Mg crystal (approximately 12 × 12 × 20 mm^3^) are shown in Figure 3b. The spectrum for the unencapsulated crystal (i.e., the crystal immersed in mineral oil) resulted in an energy resolution of 5.6% ± 0.1% (FWHM) at 662 keV, while the encapsulated crystal resulted in an energy resolution of 5.3% ± 0.1%. The relative light yield (i.e., full energy peak position) for the encapsulated crystal was also higher than that when the crystal was measured in oil (Figure 3b), suggesting better light collection efficiency for this particular crystal when encapsulated. The (non-)proportionality data in Figure 3c shows that TLYC:Ce,Mg has a relatively linear response (between 0.95 and 1.05) for a gamma-ray energy above 30 keV. Temporal data for the encapsulated TLYC:Ce,Mg is shown in Figure 3d, with measured decay constants of 64 ns ± 1 ns (4.3% ± 0.3%), 459 ns ± 1 ns (66.5% ± 0.3%), and 1.88 μs ± 0.02 μs (29.2% ± 0.4%).

### 2.4. Tl_2_LiYCl_6_:Ce,Ba

**Figure 4:**
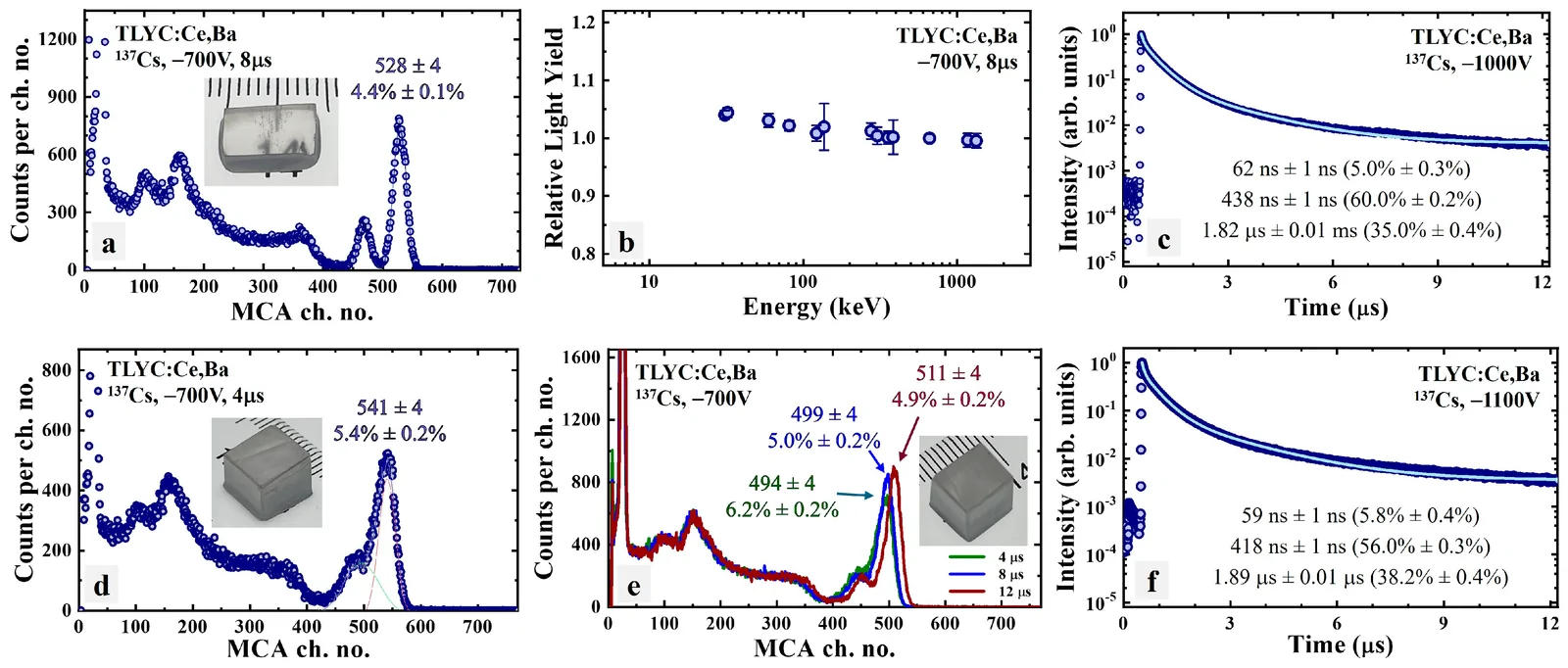
(**a**) ^137^Cs spectrum obtained with a 9 × 6 × 2 mm^3^ TLYC:Ce,Ba crystal. (**b**) Non-proportionality curve for the TLYC:Ce,Ba crystal. (**c**) Temporal data for the 9 × 6 × 2 mm^3^ TLYC:Ce,Ba crystal. (**d**) ^137^Cs spectrum obtained with a 9 × 9 × 5 mm^3^ TLYC:Ce,Ba crystal. (**e**) ^137^Cs spectra obtained with a 9 × 7 × 6 mm^3^ TLYC:Ce,Ba crystal with different shaping times. (**f**) Temporal data for the 9 × 7 × 6 mm^3^ TLYC:Ce,Ba crystal.

Figure 4a shows the ^137^Cs spectrum obtained with an approximately 9 × 6 × 2 mm^3^ TLYC:Ce,Ba crystal with 8 μs amplifier shaping time, resulting in an energy resolution of 4.4% ±0.1% (FWHM) at 662 keV. The non-proportionality curve in Figure 4b shows that TLYC:Ce,Ba has a relatively linear response for photons with an energy above 30 keV. By fitting three exponential decay functions to the temporal data shown in Figure 4c, decay constants of 62 ns ± 1 ns (5.0% ± 0.3%), 438 ns ± 1 ns (60.0% ± 0.2%), and 1.82 μs ± 0.01 μs (35.0% ± 0.4%) were calculated. A thicker TLYC:Ce,Ba crystal (approximately 9 × 9 × 5 mm^3^) collected the ^137^Cs spectrum with a 4 μs amplifier shaping time, resulting in an energy resolution of 5.4% ± 0.2% (FWHM) at 662 keV (Figure 4d). ^137^Cs spectra collected by another thick TLYC:Ce,Ba crystal of a similar thickness (approximately 9 × 7 × 6 mm^3^) with 4 μs, 8 μs, and 12 μs shaping times are shown in Figure 4e, with the best energy resolution of 4.9% ± 0.2% (FWHM) being obtained at 12 μs. Decay constants of 59 ns ± 1 ns (5.8% ± 0.4%), 418 ns ± 1 ns (56.0% ± 0.3%), and 1.89 μs ± 0.01 μs (38.2% ± 0.4%) were calculated from the temporal data of the 9 × 7 × 6 mm^3^ TLYC:Ce,Ba crystal (Figure 4f).

The comparison of ^137^Cs spectra obtained with TLYC:Ce,Ba crystals in Figure 4a,e show that, for the same amplifier shaping time, the thinner crystal (Figure 4a) resulted in a higher energy resolution compared to the thicker crystal (Figure 4e). For the thicker crystal in Figure 4e, the longer the amplifier shaping time, the better the energy resolution and the higher the full energy peak position (i.e., relative light yield).

### 2.5. Tl_2_LiYCl_6_:Ce,Ca

**Figure 5:**
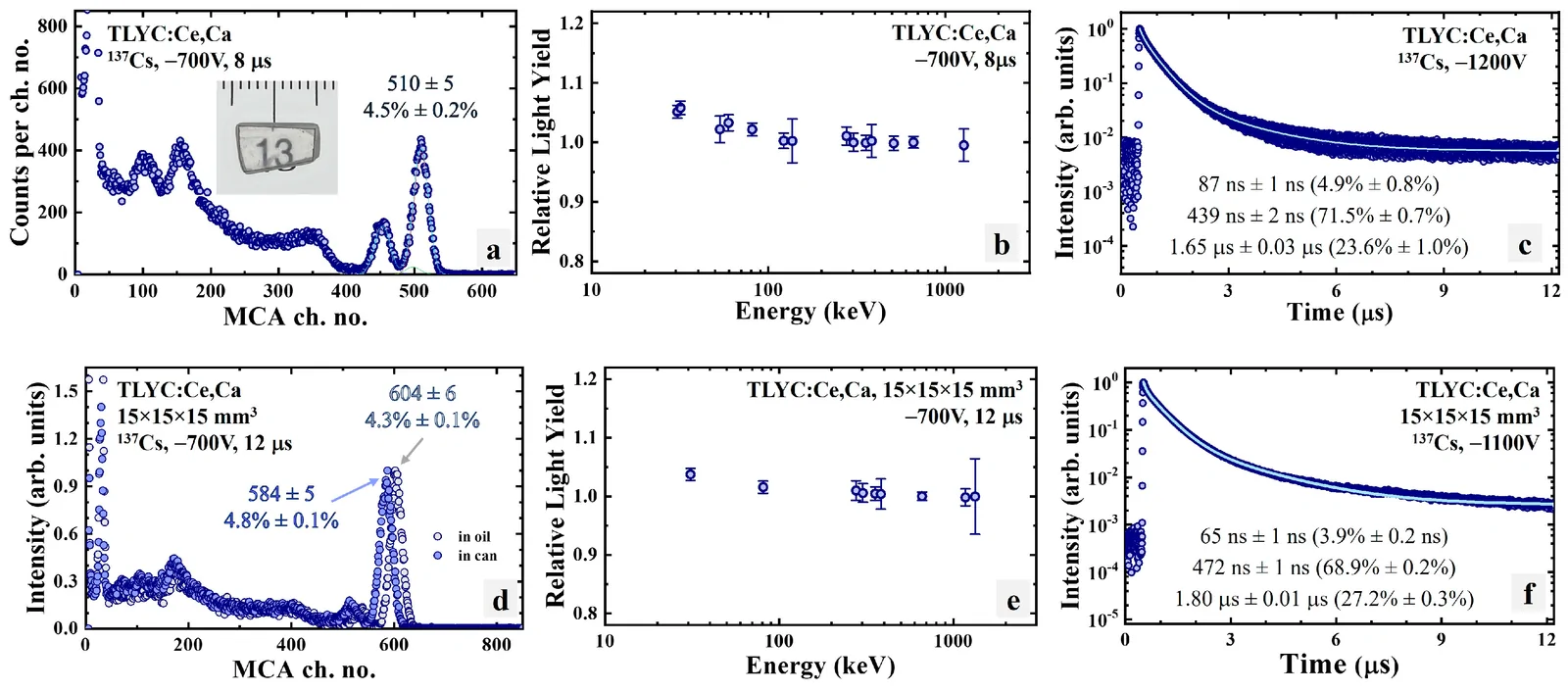
(**a**) ^137^Cs spectrum obtained with a 9 × 4 × 2 mm^3^ TLYC:Ce,Ca crystal. (**b**) Non-proportionality curve for the TLYC:Ce,Ca crystal in (**a**). (**c**) Temporal data for the 9 × 4 × 2 mm^3^ TLYC:Ce,Ca crystal. (**d**) ^137^Cs spectrum obtained with a 15 × 15 × 15 mm^3^ TLYC:Ce,Ca crystal. (**e**) Non-proportionality curve for the 15 × 15 × 15 mm^3^ TLYC:Ce,Ca crystal. (**f**) Temporal data for the 15 × 15 × 15 mm^3^ TLYC:Ce,Ca crystal.

Figure 5a shows a ^137^Cs spectrum obtained with an approximately 9 × 4 × 2 mm^3^ TLYC:Ce,Ca crystal with a 8 μs amplifier shaping time, resulting in a 4.5% (FWHM) energy resolution at 662 keV. The non-proportionality curve shows a relatively linear response to gamma-rays above 50 keV (Figure 5b). Decay constants of 87 ns ± 1 ns (4.9% ± 0.8%), 439 ns ± 2 ns (71.5% ± 0.7%), and 1.65 μs ± 0.03 μs (23.6% ± 1.0%) were calculated from the average waveforms shown in Figure 5c. Figure 5d shows the ^137^Cs spectra obtained with a thicker sample of TLYC:Ce,Ca (15 × 15 × 15 mm^3^), both as a bare crystal in oil or as an encapsulated crystal. With a 12 μs shaping time, energy resolutions of 4.3% ± 0.1% and 4.8% ± 0.1% (FWHM) at 662 keV were measured for the bare crystal in oil and encapsulated crystal, respectively. The similarity in the performance of both scintillation detector configurations, in terms of relative light yield and energy resolution, exhibited a successful encapsulation effort. The non-proportionality curve for the thicker crystal (Figure 5e) shows that it had a better linear response to gamma-rays above 30 keV. As shown in Figure 5f, the thicker TLYC:Ce,Ca crystal had measured decay constants of 65 ns ± 1 ns (3.9% ± 0.2 ns), 472 ns ± 1 ns (68.9% ± 0.2%), and 1.80 μs ± 0.01 μs (27.2% ± 0.3%), which were within the same order magnitudes and proportions to those of the thinner crystal.

**Figure 6:**
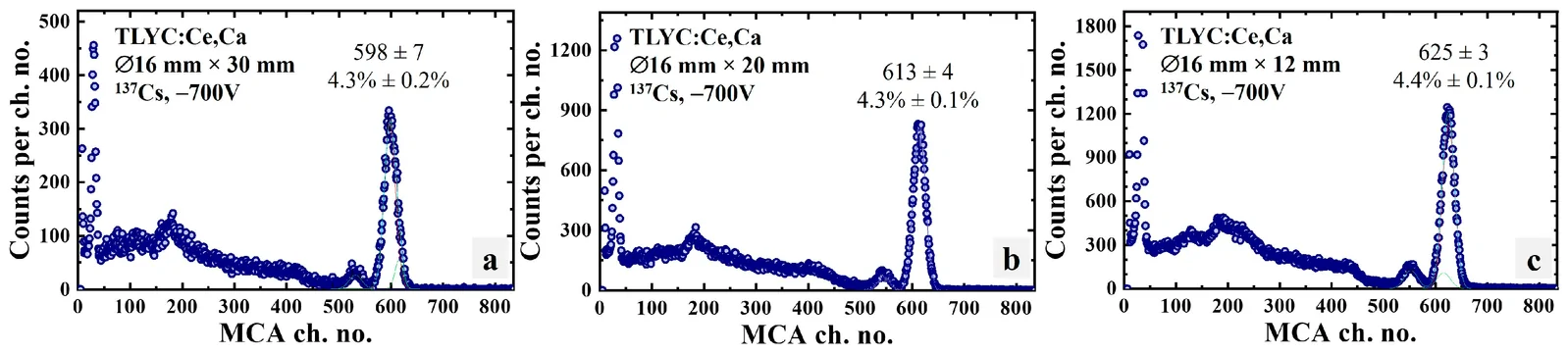
^137^Cs spectra collected with samples of TLYC:Ce,Ca measuring (**a**) ∅16 mm × 30 mm, (**b**) ∅16 mm × 20 mm, and (**c**) ∅16 mm × 12 mm.

Cylindrical samples of ∅16 mm TLYC:Ce,Ca with different sample heights were characterized, as shown by the ^137^Cs spectra obtained for each sample in Figure 6. All three samples, with 30 mm (Figure 6a), 20 mm (Figure 6b), and 12 mm (Figure 6c) crystals, performed similarly in terms of relative light yield (i.e., full energy peak position) and energy resolution. As the sample thickness became thinner (from 30 mm to 12 mm), the relative light yield improved due to better light collection efficiency.

### 2.6. Neutron Measurements: TLYC:Ce,Mg and TLYC:Ce,Ca

**Figure 7:**
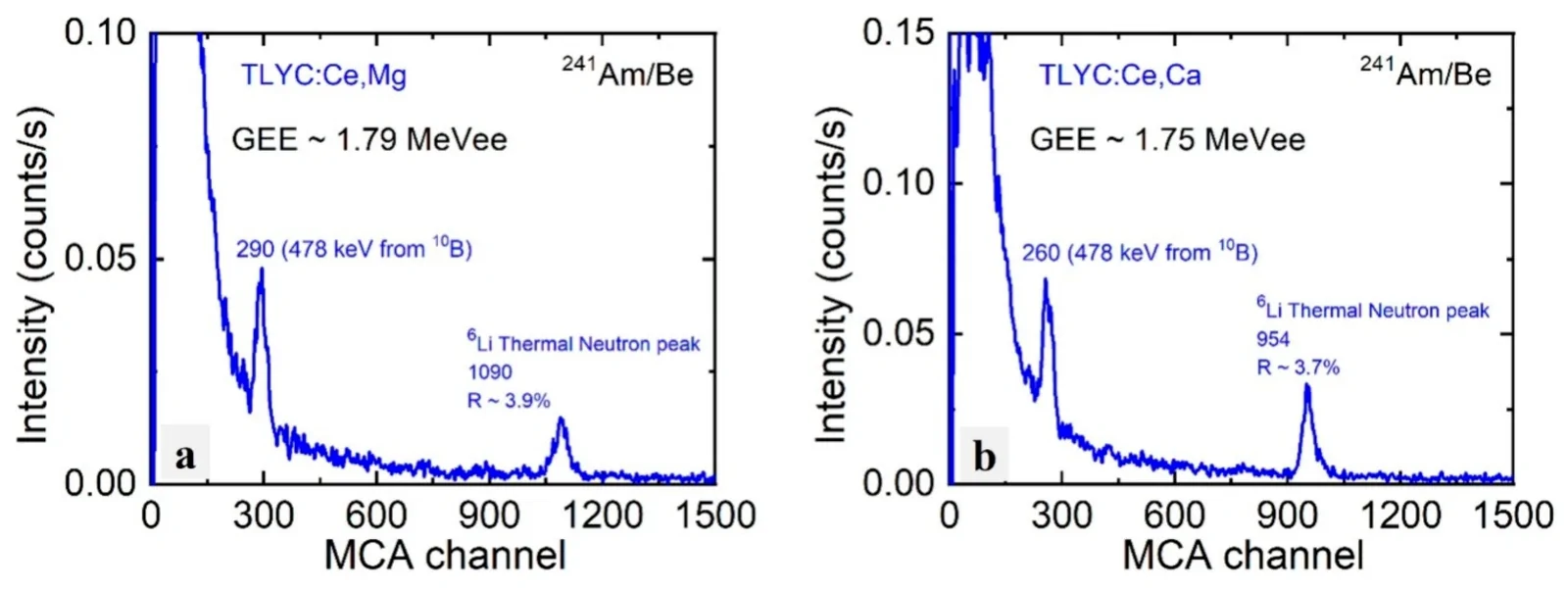
Neutron pulse height spectra measured with a moderated ^241^Am/Be source for (**a**) TLYC:Ce,Mg and (**b**) TLYC:Ce,Ca.

Thermal neutron spectra measured with a moderated ^241^Am/Be source for the Mg and Ca co-doped samples are shown in Figure 7a and Figure 7b, respectively. The gamma-equivalent energy for both samples is estimated to be around 1.8 MeVee based on the position of the 478 keV gamma peak generated by the boron shielding around the experimental setup. The thermal neutron peak is well-defined, and the energy resolution of the peak is 3.9% and 3.7% for the Mg and Ca co-doped samples, respectively.

**Figure 8:**
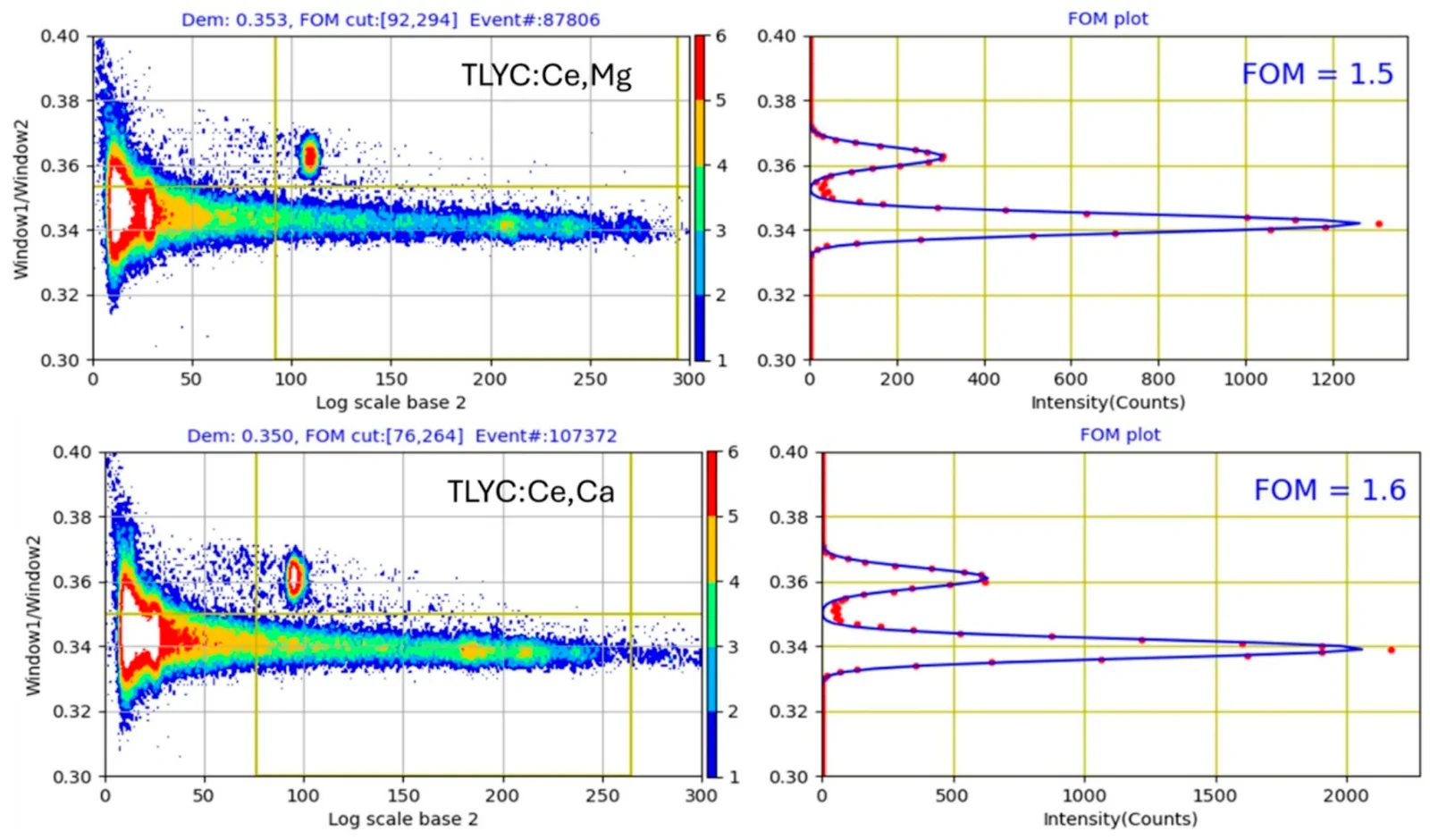
Pulse shape discrimination scatter plots collected for the samples with two different co-dopants, Mg and Ca. The PSD parameters used to create graphs were the same. The data were collected under excitation from a moderated^241^Am/Be source. FOM plots were created from PSD scatter plots, shown on right. The cuts for generating FOM plots are shown as yellow vertical lines. The numbers indicate the FOM value for each case.

Figure 8 shows the PSD scatter plots measured for both samples. The plots were created from traces collected under excitation from a moderated ^241^Am/Be source. To create the plots, each trace was processed to extract three integrals: W1, W2, and W3 (total integral). The W1 window was integrated from 0 ns to 160 ns, the W2 window from 0 ns to 400 ns and the W3 window from 0 ns to 4000 ns. The PSD factor is calculated as W1/W2 and plotted against the W3 value. The PSD plot shows the gamma band located at y = 0.34 and the thermal neutron area at y = 0.36. Both plots look quite similar, showing a clear neutron peak. The separation between gamma and thermal neutrons can be assessed using figure-of-merit (FOM), calculated as |y_neutron_ − y_gamma_|/(FWHM_neutron_ + FWHM_gamma_). FOM is created by taking a cut from the PSD scatter plot and projecting it on the Y axis. This creates a double Gaussian plot, as shown in the figure on the right. The FOM values are 1.5 and 1.6 for the Mg and Ca co-doped samples, respectively.

**Figure 9:**
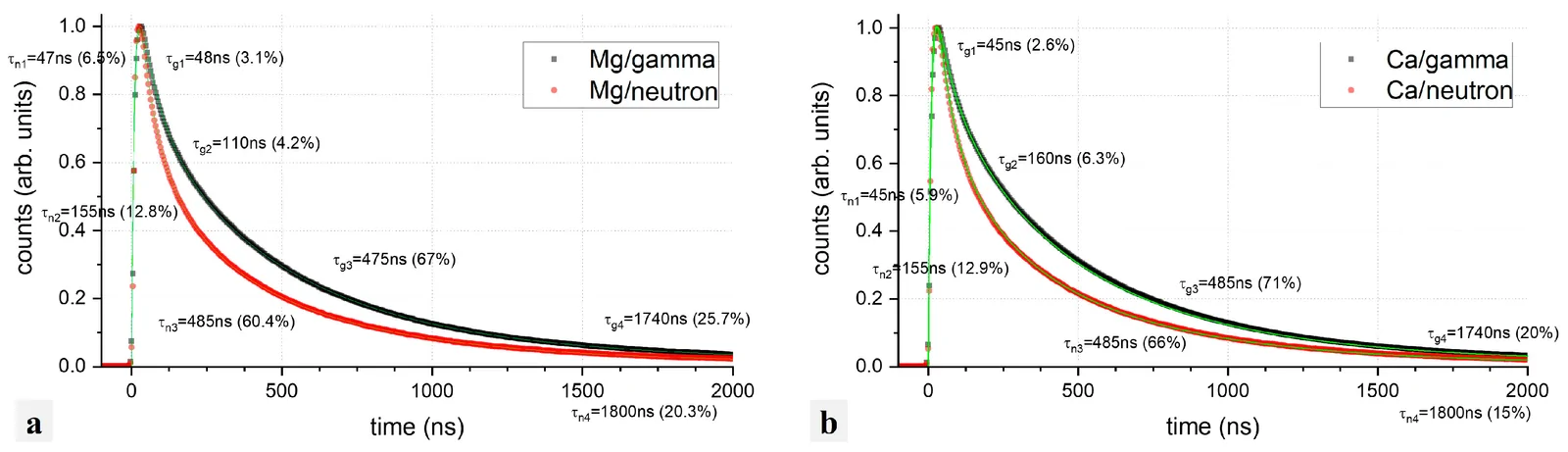
Scintillation time profiles measured for both gamma-rays and thermal neutrons for (**a**) Mg and (**b**) Ca co-doped samples.

The gamma and neutron scintillation time profiles extracted by gating on the events from the PSD plot are shown in Figure 9. The decays were fitted with four components for both samples, and the decay constants and light in each component are shown in the plots and in Table 1.

### 2.7. Tl_2_LiYCl_6_:Ce,Ca,Mg

**Figure 10:**
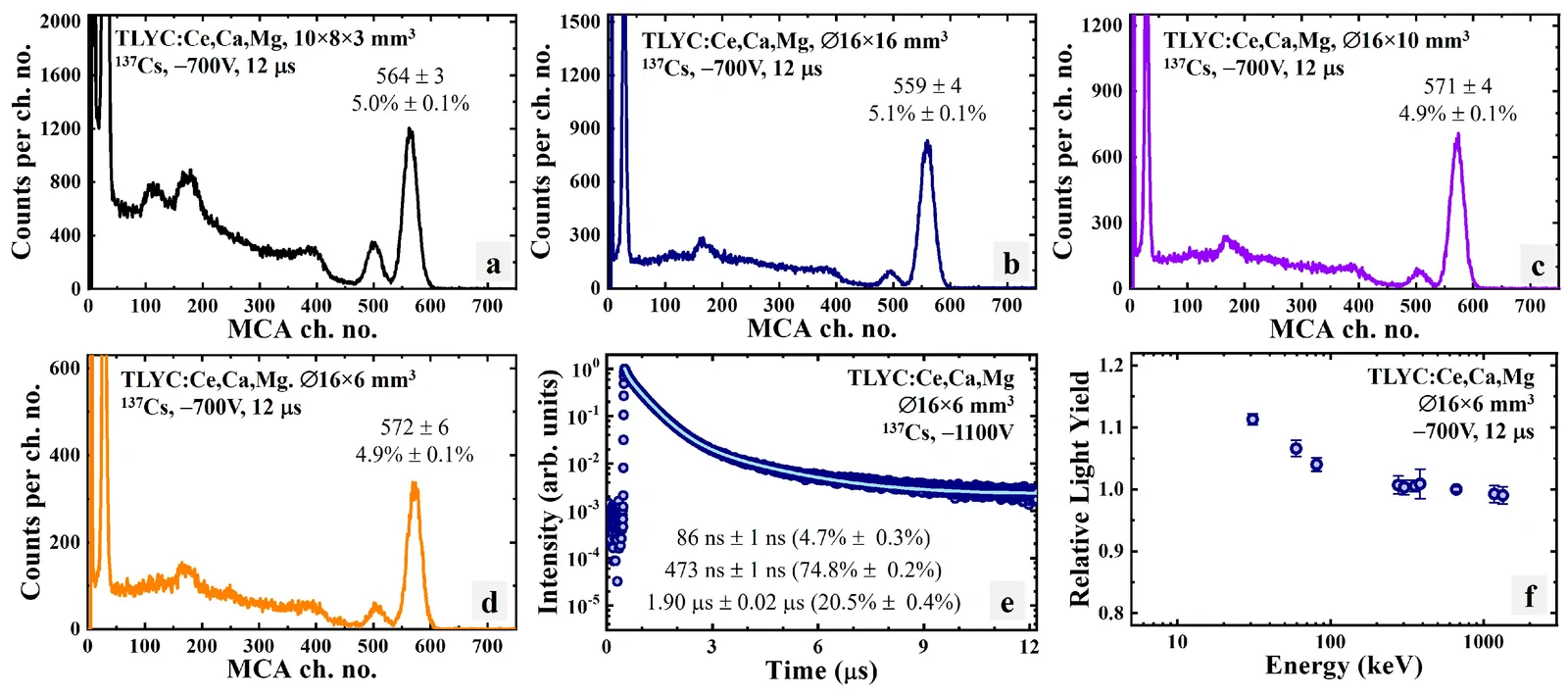
(**a**) ^137^Cs spectrum collected with a 10 × 8 × 3 mm^3^ TLYC:Ce,Ca,Mg crystal. (**b**) ^137^Cs spectrum collected with a ∅16-mm × 16 mm TLYC:Ce,Ca,Mg crystal. (**c**) ^137^Cs spectrum collected with a ∅16-mm × 10 mm TLYC:Ce,Ca,Mg crystal. (**d**) ^137^Cs spectrum collected with a ∅16-mm × 6 mm TLYC:Ce,Ca,Mg crystal. (**e**) Temporal data for TLYC:Ce,Ca,Mg. (**f**) Non-proportionality curve for TLYC:Ce,Ca,Mg.

To obtain the favorable properties of TLYC with the double dopants Ce and Ca (high relative light (i.e., peak position) and high energy resolution), as well as of TLYC with the double dopants Ce and Mg (linear response to gamma-rays), TLYC with the triple dopants Ce, Ca, and Mg was grown. The ^137^Cs spectrum obtained with a plate-like thin crystal sample of TLYC:Ce,Ca,Mg (10 × 8 × 3 mm^3^) shows an energy resolution of 5.0% ± 0.1% (FWHM) at 662 keV (Figure 10a). Similar energy resolutions and relative light yields were measured for thicker but cylindrically shaped TLYC:Ce,Ca,Mg crystals with sizes of ∅16 mm×16 mm (Figure 10b), ∅16 mm × 10 mm (Figure 10c), and ∅16 mm × 6 mm (Figure 10d). Similarity, the cylindrical crystal results indicated that the grown boule was uniform in quality and dopant distribution. Figure 10e shows the temporal data for TLYC:Ce,Ca,Mg, from which the decay constants of 86 ns ± 1 ns (4.7% ± 0.3%), 473 ns ± 1 ns (74.8% ± 0.2%), and 1.90 μs ± 0.02 μs (20.5% ± 0.4%) were calculated. Figure 10f shows that TLYC:Ce,Ca,Mg had a proportional response to γ-rays above 100 keV.

## 3. Discussion

Table 2 summarizes the results of scintillation measurements on the co-doped TLYC crystals presented in this paper and compared to TLYC results shown in Reference [17]. Based on these results, the overall apparent advantage of co-doping TLYC with alkaline earth metal ions is light yield improvements, except for co-doping with Sr^2+^ (the full energy peak in the ^137^Cs spectrum was not resolved) and Ba^2+^. It is worth noting that this improvement was obtainable even when the growths were conducted with as-received or not purified starting (raw) materials. Therefore, better energy resolution and light yield are expected with better quality chemicals.

Based on the X-ray-stimulated optical emission in Figure 1, the emission peak wavelengths for Ce-only-doped TLYC and co-doped TLYC are similar. A review of radioluminescence emission spectra in [34] of undoped and Ce-only-doped TLYC (in low concentrations) shows that the broad emission that is centered between 420 and 430 nm is likely intrinsic, though the exact nature of the scintillation processes are not known and is outside the scope of this work. The emission spectrum of TLYC:Ce,Ca shows a slightly more intense emission between 350 nm and 400 nm, which is usually attributed to Ce^3+^ emission [34]. Both emission spectra of TLYC:Ce,Mg and TLYC:Ce,Ba are more intense above the peak emission, compared to Ce-only-doped TLYC and TLYC:Ce,Ca. The primary scintillation decay time constants (400 to 480 ns) for co-doped TLYC crystals are similar in value and in fraction to Ce-only-doped TLYC, while the longest decay time constants are approximately twice those of Ce-only-doped TLYC. While the specific character of the scintillation mechanisms will be a relevant subject of study, it is beyond the scope of this work.

Divalent alkaline earth metals such as Mg^2+^, Ca^2+^, Sr^2+^ and Ba^2+^ span a wide range of ionic radii from very small to exceptionally large. Co-doping elpasolite crystals like TLYC, which has the halide–double-perovskite A_2_BB’X_6_ structure, with alkaline earth metals introduces a complex interplay of charge imbalance, ionic radius mismatches, and electronic phase changes. This occurs because alkaline earth metals are divalent (2^+^) and can substitute into elpasolite sites that are either 1^+^ or 3^+^. Such substitutions constitute aliovalent charge-compensation processes that lead to several distinct structure and electronic phenomena, such as the generation of charge-compensation defects, systematic phase transitions via ionic radius tuning, bandgap engineering and mechanical robustness and elastic properties. For example, Mg^2+^ may preferentially occupy B/B’ interstitial sites, which maximizes lattice distortions, improves optical absorption while producing a red shift, and also balances ductility. Ca^2+^ substitution at B/B’ sites introduces structural stress, and the material will become prone to precipitations or phase separations if the threshold is exceeded. Sr^2+^ may occupy A sites or B/B’ site, thereby enhancing mechanical hardness and altering carrier localization. Ba^2+^ strongly favors A-site substitution, and this drastically increases lattice volume transitions from a cubic to tetragonal symmetry. All of these co-doping profiles dramatically alter the structural Goldschmidt tolerance factor, leading to structural imperfections that affect the electronics properties and directly impact performance [35,36,37,38].

## 4. Materials and Methods

### 4.1. Crystal Growth

The growth runs of co-doped TLYC crystals described in this paper were carried out with as-received (not purified) raw materials from APL Engineered Materials, Inc. (Urbana, IL, USA) with >99.999% purity levels. The initial growth effort was simplified to allow more focus on growing single crystals with a uniform dopant distribution. The Bridgman growth method in a vertical furnace was utilized to grow all the crystals. For each growth, stoichiometric amounts of raw materials (TlCl, LiCl, YCl_3_, and CeCl_3_, and respective halide of the alkaline earth metals) were loaded into a pre-cleaned fused silica ampoule inside an inert atmosphere glove box to prevent cross-contamination and exposure to moisture. Nominal concentrations of 5% for Ce and 3% for the alkaline co-dopant were used. The loaded ampoule was then attached to a heater-vacuum system for dehydration and sealing. After sealing, the ampoule was loaded into a vertical furnace for growth.

### 4.2. Crystal Processing and Characterization Measurements

After growth, the ampoule was removed from the furnace and opened to retrieve the grown crystal boule. The boules were typically cut with a diamond wire saw to obtain the samples. The samples were then shaped and polished using Al_2_O_3_ or SiO_2_ polishing papers up to a 2000 grit. Initial measurements were carried out by placing a crystal sample inside an oil-measuring quartz cup. The purpose of this procedure was to examine the fitness of the sample prior to encapsulation. Standard NIM equipment, such as a Hamamatsu R6231-100 photomultiplier tube (PMT) (Hamamatsu City, Shizuoka, Japan), Canberra 2005 pre-amplifier (Meriden, CT, USA), and Canberra 2022 amplifier, were utilized for both gamma-ray and neutron measurements. For gamma-ray measurements, as the first initial characterization, a series of ^137^Cs spectra were obtained with different amplifier shaping times. After an optimal shaping time was determined, spectra from other gamma-ray check sources, such as ^241^Am, ^133^Ba, ^22^Na, and ^60^Co, were obtained and the information from their X-ray and full-energy peaks were utilized to determine energy resolutions, light yields, and (non-)proportionality data. Light yield was approximated by comparing the peak positions of X-ray and gamma-ray full-energy peaks, as well as by taking into account the emission spectrum of co-doped TLYC crystals and NaI:Tl [39], the quantum efficiency or spectral response of the PMT [40], and the light yield of the NaI:Tl crystal (38,000 ph/Mev) provided by the vendor [39]. To determine decay times, anode signals from the PMT were digitized and collected using CAEN DT5720 (Viareggio, Lucca, Italy) and then analyzed off-line. Averaged waveforms from the full-energy peak area were analyzed in OriginLab^®^ 2019 (Northampton, MA, USA) through various iterations using the ExpDec3 three-term exponential decay function.

Two encapsulated co-doped crystals, TLYC:Ce,Mg and TLYC:Ce,Ca, were sent to Radiation Monitoring Devices, Inc. (RMD, Inc., Watertown, MA, USA) for measurements with a moderated or thermalized 340 mCi americium–beryllium (^241^AmBe) neutron source. For testing, each encapsulated crystal was coupled to a Hamamatsu R6233-100 super-bialkaline PMT and associated NIM equipment. Gamma-ray characterization using a ^137^Cs check source and the collection of energy-equivalent Am-Be spectra were performed for several amplifier shaping times. With the PMT biased at −1200V, the anode signals were collected with a CAEN DT5720 digitizer. The waveforms were analyzed off-line to determine the pulse shape discrimination (PSD) of the gamma and neutron events and calculate the Figure-of-Merit (FOM), which quantitatively measured the separation between gamma and neutron events.

The X-ray-excited optical luminescence (XEOL) spectra of co-doped TLYC crystals were collected using a 30 keV X-ray tube source. The emission spectra were collected by a fiberoptic-coupled Ocean Optics spectrometer that employed a silicon CCD readout.

## Figures and Tables

**Table 1:** Decay constants extracted from the PSD events for TLYC:Ce,Mg and TLYC:Ce,Ca.

Crystal	γ Decay Constants	n Decay Constants
TLYC:Ce,Mg	48 ns (3.1%), 110 ns (4.2%), 475 ns (67%), 1.74 μs (25.7%)	47 ns (6.5%), 155 ns (12.8%), 485 ns (60.4%), 1.8 μs (30.3%)
TLYC:Ce,Ca	45 ns (2.6%), 160 ns (6.3%), 485 ns (71%), 1.74 μs (20%)	45 ns (5.9%), 155 ns (12.9%), 485 ns (66%), 1.8 μs (15%)

**Table 2:** Summary of co-doped TLYC crystal scintillation properties.

Crystal	Energy Resolution at 662 keV (%, FWHM)	Estimated Light Yield (ph/MeV)	γ Decay Constants	FOM
TLYC:Ce	3.9	25,000	56 ns (3%), 432 ns (65%), 923 ns (32%)	2.4
TLYC:Ce,Mg	4.7	28,100	64 ns (4.3%), 459 ns (66.5%), 1.88 μs (29.2%)	1.5
TLYC:Ce,Ca	4.3	28,900	87 ns (4.9%), 439 ns (71.5%), 1.65 μs (23.6%)	1.6
TLYC:Ce,Sr	Not resolved	-	84 ns (7.1%), 327 ns (71.0%), 1.43 μs (21.9%)	-
TLYC:Ce,Ba	4.4	24,200	59 ns (5.8%), 418 ns (56.0%), 1.89 μs (38.2%)	-
TLYC:Ce,Ca,Mg	4.9	26,500	86 ns (4.7%), 473 ns (74.8%), 1.90μs (20.5%)	-

## Data Availability

The datasets presented in this article are not readily available because of proprietary reasons. Requests to access the datasets should be directed to the Corresponding Author.
